# *Erratum:* Vol. 72, No. RR-2

**DOI:** 10.15585/mmwr.mm7243a4

**Published:** 2023-10-27

In the *MMWR* Recommendations and Reports, “Prevention and Control of Seasonal Influenza with Vaccines: Recommendations of the Advisory Committee on Immunization Practices — United States, 2023–24 Influenza Season,” on page 15, in the first paragraph, the second sentence should have read, “Examples include AS01B (in Shingrix, recombinant zoster subunit vaccine) (*146*), AS01E (in **Arexvy**, respiratory syncytial virus vaccine) (*147*) MF59 (in Fluad Quadrivalent [aIIV4]) (*56*), and cytosine phosphoguanine oligodeoxynucleotide (in Heplisav-B, a recombinant hepatitis B surface antigen vaccine) (*148*).” In addition, on page 25, reference 147 should have read, “**Arexvy** [Package insert]. Durham, NC: GlaxoSmithKline; 2023.”

